# Foreign Correspondence

**Published:** 1872-04

**Authors:** H. Tombocken

**Affiliations:** Vienna


A MONTHLY RECORD OF

*Medicine, Surgery and the Collateral Sciences.*

EDITED BY J. ADAMS ALLEN, M.D., LL.D.; AND WALTER HAY, M.D.

VOL. XXIX. — APRIL, 1872. — NO. 4.

### Foreign Correspondence.

VIENNA, February 29, 1872.

DEAR DOCTOR:

A few days ago we received a copy of the Chicago Evening Post, containing the notice of the commencement exercises of Rush Medical College, with the usual number of graduates. By the time I return to Chicago, I expect to see the "Old Rush" rebuilt and in full operation again, to welcome all who may seek knowledge within its walls.

You will remember I told you that I was going to Prague; so I did. Upon arriving in that city I immediately went to the Hospital College buildings. Making inquiries concerning the lectures, etc., I discovered that Prague was, at present, a "one-horse" concern. Stating my opinion to Dr. Walenta who is now residing in that city, the doctor admitted its truth, and could not conscientiously ask me to stay in Prague.

In 1848 the University of Prague was in its glory; since then its light has waned, and now only flickers.

Whilst in Prague I became acquainted with Dr. Tiefdrung, physician to ex-Emperor Ferdinand. The duties of the physician are, to visit the ex-monarch three times a week; in case of sickness he may be required to remain through the night. The remuneration consists in free dwelling in the castle, fuel, and 1,600 florins ($800) per annum.

After seeing the sights of Prague, I started for Vienna. Freeing myself from the clutches of a hackman, I arrived at the General Hospital in which the lectures and clinics are conducted. It is an immense structure, enclosing and dividing a large space of ground into several gardens or yards, the largest of these being about the size of a Chicago block.

The building contains over 100 wards of about 40 x 80 feet each, with 2,500 beds. There are sixty single rooms for patients that are able to pay for the superior accommodations therein offered. About 25,000 house patients are treated annually.

Connected with the General Hospital is the lying-in hospital for Lower Austria. A part of this is used for the education of midwives; the larger part, in which about 6,000 births take place annually, for the medical student. Adjacent, is also the hospital for the insane.

The Faculty is composed of regular (ordinary) professors, and irregular (extraordinary) professors and docents, the latter being for the greater part assistants to the professors, and the whole forming a small army.

A systematic course of lectures is given only by the following professors: Prof. Hyrtl, anatomy, descriptive, topographic. Prof. Brucke, physiology. Prof. Rokitansky, pathology. The other lectures are delivered by some of the docents.

The following professors simply conduct their respective clinics: Prof. Braun, obstetrics and diseases of women; Billroth and Dumreicher, surgery; Hebra, dermatology; Arlt and Jaeger, ophthalmology; Duchek, medical clinic. (The vacancies produced in the medical clinics by the death of Oppolzer, and resignation of Skoda, are at present only temporarily filled.) Profs. Sigmund and Zeissl, syphilis. Each of the above professors has his corps of assistants.

Other clinics, as for chronic nervous diseases, diseases of the larynx, chest, etc., to be treated by special means, as electricity, inhalation, etc. Also, two clinics for diseases of the ear, conducted by Profs. Gruber and Pollitzer.

The number of students and doctors attending the hospital is probably between 1,000 and 1,500; I could not inform myself of the exact number. A large proportion of these are foreigners, i. e., non-residents of Austria; and America furnishes its share of the latter. Two young ladies—one an American, the other a Prussian—are also attending, and seem to feel quite at home among the young men.

What makes Vienna so attractive to foreigners? It cannot be its great men, for from them you derive little or no benefit. Take, for instance, the great Rokitansky, the father of pathology, who is favorably known wherever pathology is studied and taught; he is neither a lecturer nor teacher, his class is small, composed of those students who are obliged to get his ticket in order to graduate. You cannot understand a word of his lecture unless you are within four or five feet of his chair. One of his assistants gives short courses on pathology, but it is hurried over so, that you simply have specimens—often rare ones, it is true—shown to you with a meagre description. The practical department also lacks a good teacher, and were a "lesser" in the place of the "great," it would be all the better for those who come to learn. "Material" is, of course, abundant, for this as well as other purposes.

Prof. Braun, of the lying-in department, is no lecturer and not much of a teacher, although a thoroughly competent man to attend to his patients, practically.

Hebra, the great dermatologist, simply conducts his clinic and occasionally gives vent to his humor at the expense of his patients, which is more amusing than instructive.

Hyrtl, the anatomist, is certainly the most popular among the professors, if I am to judge by the number of his hearers. He is a fluent, agreeable lecturer, as well as a good instructor. An acquaintance thinks him equal to Prof. Ford, of Ann Arbor.

The amount of material for clinical purposes is, of course, large, and is used (and abused) freely and to good advantage so far as the student is concerned.

Syphilitic and skin disease patients are brought into the lecture room without even the fig leaf. The females are also fully exposed, and obliged to allow all that may wish, to examine them.

At the lying-in hospital, patients are received every evening. The assistant examines them for the purpose of ascertaining their condition, then about twenty students will each, in turn, examine the same woman, she being obliged to submit. From the time of her admission until her confinement, she may be called upon several times to allow herself to be examined for purposes of instruction.

For the parturient room twelve students are allotted to act as practicant (practitioner), this number being changed every twenty-four hours, thus giving the students an opportunity to obtain a practical knowledge of obstetrics. *Some* examining is done, of course. If a case of unusual interest occurs, it is reserved until the lecture hour and brought before the class, then delivered of her fruit either by manual or instrumental assistance as the case may demand.

A peculiar, and perhaps the most attractive feature of this institution consists in the so-called private courses. These courses are generally given by the docents, are from five to eight weeks in duration, being twenty-five to forty hours, one hour each day, and are given upon almost every subject connected with medicine.

To illustrate : upon percussion and auscultation, laryngoscopy, ophthalmoscopy, electrotherapeutics, examination of urine, hydrotherapeutics, syphilis, surgical operations, bandaging, operations on the eye, obstetrical operations, etc., etc. The courses are thoroughly practical, and mostly intended only for the advanced student or physician.

A clinic for diseases of children is given at St. Anna's Children's Hospital, being divided into medical and surgical. Also, a clinic for diseases of infancy (as old as two months,) at the Foundling's Home.

At the General Hospital, mercury is very freely used, chiefly by inunction, for the treatment of syphilis, whereas at another hospital it is discarded altogether, the physicians of the latter asserting that mercury produces all those difficulties usually ascribed to syphilis.

Whilst Hebra is a unicist, the professors of the syphilitic department proper are dualists in reality, although they acknowledge but one poison, the syphilitic, which produces the chancre ; but will not recognize a chancroid, "it either is, or is not, a chancre," they don't want things mixed, the chancroid of the French being called a foul ulcer that may be produced by the inoculation of pus taken from any ulcer that has been irritated. Syphilization is not countenanced.

Dr. Lastorfer, of this city, has for the last eight months been engaged in examining the blood of syphilitics, and found that by placing a drop on a microscopic slide, keeping this in a moist chamber, in about twenty-four hours a hyaline body, round, one-third the diameter of the blood globule, would be developed, whilst the blood of non-syphilitics would in no case show this; also, that these bodies became, less as the cure progressed under the proper treatment. But now came Prof. Hebra, and said that he had found these bodies in the blood of variola patients. Then came Prof. Wedl: he had found them in the blood of almost all persons; and that which promised much was blown to the winds.

To-day I heard that Dr. Bumstead, of New York, is visiting the hospital, but as I do not know him I may have met him without knowing it.

In about three weeks I expect to leave here. The summer I intend to spend in Berlin, the course being little more than two months in duration. Should I find anything of interest in Berlin I will write.

Yours, H. TOMBOCKEN.

